# Lectures on Diseases of the Skin

**Published:** 1887-02

**Authors:** Henry Wile

**Affiliations:** Lecturer on Dermatology, Atlanta, Ga.


### LECTURES ON DISEASES OF THE SKIN.

---

DELIVERED AT THE ATLANTA MEDICAL COLLEGE DURING THE SESSION OF 1885–6, BY HENRY WILE, M. D., LECTURER ON DERMATOLOGY, ATLANTA, GA.

---

### LECTURE XII.

---

ECZEMA CONTINUED—ETIOLOGY—PATHOLOGY—DIAGNOSIS—PROGNOSIS—TREATMENT, INTERNAL.

---

Reported Stenographically for THE ATLANTA MEDICAL AND SURGICAL JOURNAL by W. Kay Tewksbury.

---

The etiology of eczema is as complex as the symptomatology, the causes being so manifold and diverse. There can be no doubt as to the existence of a constitutional predisposition which renders certain individuals especially liable to the disease. This predisposition consists in a weakness on the part of the cutaneous tissues to withstand irritations of various kinds, both direct and reflex, and it is hereditary in some cases. The disease itself is not hereditary, nor is it contagious. It is not infrequently found associated with internal diseases, as Bright's disease, diabetes mellitus, asthma, rheumatism, gout, disorders of the alimentary canal and uterine affections; but a casual relation is by no means established. That an intimate relation may exist was made clear to me in several cases. In one case the patient, an elderly lady, suffered from asthma and an eczematous eruption on the hands. It was repeatedly noticed that when the asthmatic paroxysms occurred the eruption almost disappeared, and when the paroxysms subsided the eczematous lesions became aggravated. In another case the patient, a middle-aged man, had an eczematous eruption, involving the scrotum and lower extremities, associated with a chronic intestinal catarrh. It was apparent that when the intestinal discharge moderated the cutaneous symptoms became worse, and conversely. The relation between the internal and external disease in these cases was not that of cause and effect. The affection of the skin did not appear to be symptomatic of the internal disorder any more than the latter was symptomatic of the former, but both seemed to the effect of a common cause operating in a constitution predisposed to catarrhal inflammation.

In females eczema is at times encountered during pregnancy and the period of lactation, and the chronic forms of the disease seem occasionally to be influenced by menstruation.

The external causes are numerous and consist of agencies by means of which the skin is irritated. Among these is a large class of medicinal preparations and chemicals, such as mustard, tincture of arnica, croton oil, turpentine, cantharides, corrosive sublimate, iodine, carbolic acid, mineral acids and alkalies. The last, when present in undue percentage in soap, constitute a common cause. Then again constant exposure of certain parts, especially the hands and arms, to irritating material, necessitated by employment in the various industries, must be mentioned. Thus, water among washwomen, dyestuffs among dyers, irritating chemical materials among electro-platers and typers. The wearing of under-clothing dyed with aniline colors may provoke an eruption. It occurs from exposure to extremes of temperature, whether to the direct rays of the sun, common among masons, or to the fire of a furnace, as among firemen, causing erythematous eczema, or to the sudden cold changes of winter, that gives rise to fissured eczema of the hands and lips. Morbid discharges from the ears, nose, genitalia and bowels, also the perspiration in the axillæ and about the thighs in corpulent individuals, are fruitful causes of local eruptions, where cleanliness is not observed.

Eczema is often found associated with other cutaneous diseases, not merely as a coincident, but a complication or effect. Thus, in scabies, pruritus, pediculosis and other affections that occasion itching, the scratching on the part of the patient is often so severe as to produce a decided inflammation which may develop into eczema. Likewise, long-continued irritation from rough wearing of apparel, bandages, trusses, etc., may mechanically produce the disease.

Eczema is observed among all classes and conditions, rich and poor, old and young, male and female. It occurs more frequently in the earlier periods of life, and statistics show that it is more common among males.

The pathology of acute eczema is much the same as that of any inflammatory process resulting in the development of papules, vesicles and pustules. In papular eczema the tissue of the papillæ is swollen and contains a cellular exudate which extends up through the rete mucosum and appears there composed of spindle-shaped cells forming a network. The cells of the rete are also granular. In vesicular eczema there is a circumscribed serous exudation beginning between the rete and papillary layers. The exudation contains, besides serum, some spindle-shaped cells and cellular débris. As the exudation inreases the epidermal layers grow thin and tense until the vesicle finally ruptures and its fluid contents ooze out upon the surface. Where the process continues for a time the newly formed epidermis remains thin and the exudation, instead of forming vesicles, filters through the thin epidermis directly upon the surface—hence the weeping surface of eczema. In pustular eczema the lesions are the result of a further development of the vesicles, their contents, becoming rich in cells, undergo fatty degeneration with the formation of pus. The pustules may also be due to a direct fatty degeneration of a collection of cells.

The changes wrought in the structure of the skin by the continuation of an eczematous inflammation are striking. The most evident appearance is a marked thickening of the skin, due to an enlargement of the individual papillæ, hypertrophy of the layers of the epidermis and the connective tissue of the corium. There is also to be seen a deposit of pigment in the lower layers of the rete mucosum, and stripes of pigment in the corium, which latter are regarded by some as the residue of obliterated blood-vessels. The blood-vessels are surrounded with cell-growth, the lymphatics may be seen dilated, amkulla-like, the hairs are atrophied and rest loosely in the follicles, while the sebaceous and sweat glands are also atrophied from the long-continued pressure of the hypertrophied connective tissue of the corium.

In the diagnosis of eczema one will almost invariably be guided by an impression which the cutaneous symptoms convey. Certain features and characteristics are always present that serve to stamp the process as eczematous. This impression, if analyzed, may be resolved into four elements:

1. Eczema is an inflammatory disease.

2. Its lesions are multiform, changeable, occur without special arrangement and have a tendency to diffuse themselves.

3. Its course of development in typical cases is striking, being characterized by *congestion*, the beginning, *effusion or oozing of serum*, the acme, and *desquamation*, the end of the process.

4. Itching is invariably present, and in most cases it is troublesome, even distressing to the patient.

There are some diseases, however, with which it may be confounded, and the differential diagnosis will, in such event, rest upon special differences between the diseases. Thus, on the scalp it may be mistaken for seborrhœa, but the latter disease involves more or less the whole surface of the scalp, especially the vertex and parietal regions, and is seldom accompanied by inflammatory symptoms. The surface of the scalp is pale, anæmic and covered with a varying thickness of oily scales. In addition this itching, when present, is slight, whereas in eczema of the scalp the skin is the seat of marked inflammatory changes. It is more or less confined to one region and occurs in variously-sized, isolated spots, from which there may be oozing, resulting in the development of thin, dry crusts. Besides this, where seborrhœa continues for a time, the hairs become dry and come out or break off. In eczema the hairs are tight and do not come out, except where the disease has existed for a long time. In acute eczema the hairs are tight.

It may be mistaken for psoriasis, especially when the latter disease occurs on the scalp. Both are inflammatory diseases, but the lesions of psoriasis are sharply defined, whereas in eczema they shade off gradually in the surrounding skin. Then again the scales in psoriasis are fine, imbricated, bran-like, occurring in more or less thick lamellæ, whereas in eczema the scales are thin, dry, and do not accumulate to any marked extent. Then again in psoriasis, when the scales are removed, we have bleeding points which is not the case in eczema.

Eczema is distinguished from favus, a parasitic disease mostly of the scalp, by the absence of certain features which especially characterize the latter disease. One of these features consists in the presence of favus cups, pin-head to pea-sized, hard, round bodies, having a central depression and composed of a vegetal fungus, epidermal scales and foreign matter. Favus being a parasitic disease, microscopic examination can always settle the question of diagnosis.

Eczema may resemble scabies by the presence of a variety of lesions, but here the absence of the *burrow* would eliminate the latter disease. The parts attacked by scabies are peculiar; beginning with the interdigital spaces it extends on the flexor surfaces and especially invading the genital regions and buttocks. The itching of eczema occurs at any time, day or night, whereas the itching in scabies, which may be just as distressing, occurs especially at night after the patient retires.

Vesicular eczema may be confounded with herpes zoster, but the latter affection is attended with prodromal neuralgic pain, and the vesicles occur in groups along the course of the cutaneous nerves in certain regions of the body. The lesions also exhibit a tendency to remain intact throughout the whole course of the disease, while in eczema they rupture soon after their development.

Eczema is distinguished from syphilitic cutaneous disease by the symptom of itching, which is rarely present in syphilis, by the serous catarrhal discharge from the surface, by the superficial character of the disease, by the absence of induration, ulceration and destruction of tissue, and by the general diffuse character of its lesions. In the erythematous forms of syphilis, or roseola, the spots do not disappear under pressure on account of a slight hemorrhage from the blood-vessels, whereas in eczema the spots disappear.

The differential diagnosis between squamous eczema of the palms of the hands or soles of the feet and palmar or plantar syphiloderm may present some difficulty, and it may even be necessary to study the case to find out the course of development of the process before arriving at a positive conclusion. Yet in most cases the absence of induration and of a tendency of the lesions to assume a crescentic shape, together with the history, is sufficient for decision. Syphilis resembles eczema when appearing in the form of fissures about the mouth or arms. Still, here close observation will show that the edges of the fissure of eczema are sharp like those of a clean cut, whereas the edges of the fissure of syphilis are uneven or rounded on account of the infiltration that is always present.

Not infrequently erysipelas is mistaken for acute eczema. But the latter disease never exhibits that œdema and swelling which is so characteristic of the former disease. Besides that, in erysipelas the lesion starts from a single point, is sharply defined and the discharge takes place from bullæ or large blebs, whereas in eczema the process usually starts from several points, the lesions shade gradually into the surrounding tissue and the discharge takes place from numerous ruptured vesicles.

Another disease with which eczema may be confounded is scarlatina, especially when the former occurs in the erythematous form. Scarlatina is an acute eruptive disease of childhood, and is always accompanied by marked constitutional disturbances, such as an elevation of temperature and throat symptoms, which do not occur in eczema.

The prognosis is always favorable, except when the cause is some incurable organic disease, or when there is present an hereditary element. It varies according to the extent, locality and duration of the disease. Small, localized spots are usually more obstinate to treatment than large or more diffuse lesions, and about the hands, the orifices of the body and on the scrotum, eczema is as a rule rebellious on account of the liability to frequent relapses. The disease may be said to be curable, but the prognosis should be guarded as to duration of treatment on account of the obstinacy of some forms, and as to the permanency of the cure on account of the proneness of the disease to recur.

In the treatment of eczema it must first be determined whether the process is acute or chronic, as the methods employed in each case are almost diametrically opposed. The general principle underlying the methods of treatment of acute eczema is that of allying irritation, while the general principle governing the management of chronic eczema is that of creating irritation. There is no sharp line of demarcation between these two forms, because the one runs into the other so gradually. Acute eczema is distinguished as that form in which there are acute symptoms, a bright red color, hot feeling of the surface, and in which the disease develops and disappears in a short period of time· Whereas the disease is regarded as chronic after it has existed for a time and developed into a certain sub-acute condition in which the inflammatory process is carried on on a low scale. Internal and external treatment are both used, but the latter is by far the most efficacious and, in most cases, alone necessary for a cure.

The internal administration of remedies is more especially indicated when the disease stands in a causal relation or association with some systemic disturbance. In the acute varieties when there exists any disorder of the alimentary canal, such as constipation, it is well to administer an aperient of some kind—a Seidlitz powder or a tablespoonful of Rochelle salts in a glass of water before breakfast. The following, known as *mistura ferri acida*, is a valuable combination:

℞. Magnesii sulphatis........................... ʒvi

Ferri sulphatis................................ grviii

Acidi sulphurici diluti.......................... ʒi

*M.* Infusi quassiæ................................ ℥iv

*Sig.*—One tablespoonful in a half glass of water before breakfast.

This medicine as a purgative is given in tablespoonful doses before meals and diluted in water. It is given once or twice a day, according to the habit of the patient; where a tonic action on the bowels is desirable it may be given in teaspoonful doses after meals, well diluted. To children it may be given in doses of from one to two teaspoonfuls.

In infants, when the disease is reflexly associated with indigestion or dentition, occasional doses of castor oil or olive oil are of great service. In many instances some one of the various aperient mineral waters is sufficient. In chronic eczema, arsenic is of value, especially in the squamous variety. It should, however, never be administered where there is any gastric or intestinal disorder. It may be given in the form of Fowler's solution. Small doses are preferable, from one to three minims diluted in water three times daily after meals, or a thirtieth of a grain of arsenious acid. These doses should be continued for a long period before good results can be obtained. The effect of the remedy must also be carefully watched and toxic symptoms avoided. Arsenic may be prescribed in combination with wine of iron, thus acting happily in anæmic conditions. It is also prescribed with the alkalies, such as liquor potassa or acetate of potash, where there is a gouty or rheumatic tendency, or where a diuretic effect is desirable.

In chronic eczema, where there is any general debility or weak constitutional condition due to malnutrition or scrofulous habit, it s well to prescribe cod liver oil, especially in children. Its use should also be extended over a long period of time. In infants, when the disease exhibits a tendency to relapse frequently, calomel in doses of 1/8 to 1/4 grain three times daily for several days has been observed to exert a favorable influence. Any disorder of the stomach or bowels, any co-existent internal disease must receive appropriate treatment, as the correction or amelioration of such effects for good the cutaneous systems. The diet should be carefully regulated whenever there are symptoms of indigestion, and such articles of food are to be interdicted that are apt to give rise to gastric irritation otherwise food ; has little or no influence upon the development of the disease.

